# **Association between Obesity and Cardiometabolic Health Risk in Asian-Canadian Sub-Groups**

**DOI:** 10.1371/journal.pone.0107548

**Published:** 2014-09-15

**Authors:** Jason X. Nie, Chris I. Ardern

**Affiliations:** School of Kinesiology and Health Science, York University, Bethune College, Toronto, Ontario, Canada; McMaster University, Canada

## Abstract

**Objectives:**

To quantify and compare the association between the World Health Organizations’ Asian-specific trigger points for public health action [‘increased risk’: body mass index (BMI) ≥23 kg/m^2^, and; ‘high risk’: BMI ≥27.5 kg/m^2^] with self-reported cardiovascular-related conditions in Asian-Canadian sub-groups.

**Methods:**

Six cycles of the Canadian Community Health Survey (2001–2009) were pooled to examine BMI and health in Asian sub-groups (South Asians, Chinese, Filipino, Southeast Asians, Arabs, West Asians, Japanese and Korean; N = 18 794 participants, ages 18–64 y). Multivariable logistic regression, adjusting for demographic, lifestyle characteristics and acculturation measures, was used to estimate the odds of cardiovascular-related health (high blood pressure, heart disease, diabetes, ‘at least one cardiometabolic condition’) outcomes across all eight Asian sub-groups.

**Results:**

Compared to South Asians (OR = 1.00), Filipinos had higher odds of having ‘at least one cardiometabolic condition’ (OR = 1.29, 95% CI: 1.04–1.62), whereas Chinese (0.63, 0.474–0.9) and Arab-Canadians had lower odds (0.38, 0.28–0.51). In ethnic-specific analyses (with ‘acceptable’ risk weight as the referent), ‘increased’ and ‘high’ risk weight categories were the most highly associated with ‘at least one cardiometabolic condition’ in Chinese (‘increased’: 3.6, 2.34–5.63; ‘high’: 8.9, 3.6–22.01). Compared to normal weight South Asians, being in the ‘high’ risk weight category in all but the Southeast Asian, Arab, and Japanese ethnic groups was associated with approximately 3-times the likelihood of having ‘at least one cardiometabolic condition’.

**Conclusion:**

Differences in the association between obesity and cardiometabolic health risks were seen among Asian sub-groups in Canada. The use of WHO’s lowered Asian-specific BMI cut-offs identified obesity-related risks in South Asian, Filipino and Chinese sub-groups that would have been masked by traditional BMI categories. These findings have implications for public health messaging, especially for ethnic groups at higher odds of obesity-related health risks.

## Introduction

Asians currently represent the fastest growing ethnic group in Canada, with South Asians (4.0% of total Canadian population) and Chinese (3.9%) currently ranked as the first and second largest visible minority groups, respectively [1]. Statistics Canada has projected that by 2031, the visible minority population in Canada could increase to 14.4 million people, more than double the 5.3 million reported in 2006 [2]. The largest contributors to this increase are the South Asian population, which is expected to increase 3-fold from 1.3 million in 2006 to 4.1 million in 2031, while the Chinese population is projected to grow from 1.3 million to 3 million [2].

Our knowledge of obesity and cardiometabolic health risks has been historically derived from studies of Occidental groups or persons of White European or American ancestry, the assumptions of which may not hold true when applied to other ethnic groups [3], [4]. Despite having a lower prevalence of obesity, Asians are known to be at an increased risk of cardiovascular disease (CVD) risk factors compared with those of European descent [4]–[9], a finding that has been attributed at least in part due to differences in body fat distribution and body build and frame size [10]–[14]. Moreover, CVD risk factors associated with excess weight also vary by Asian sub-groups [4], [7], [15]–[20]. For example, despite lower mean body mass index (BMI), Asian Americans are 30–50% more likely to have Type 2 Diabetes Mellitus (T2DM) than their White counterparts (6). In this analysis, Asian Indians had the highest odds of prevalent type 2 diabetes, followed by Filipinos, other Asians, and Chinese [6]. Insulin resistance has also shown to be higher in Asian Indians, and higher prevalence of metabolic syndrome is seen among Filipino and Japanese compared to other Asian groups [8], [21]–[23].

Given that health risk associated with a given level of adiposity has been shown to be higher in Asians when compared with White Europeans and Americans, the use of conventional BMI cut-off points of 25 kg/m^2^ (overweight) and 30 kg/m^2^ (obesity) may underestimate the prevalence of obesity and its associated health risk [3], [9], [11]. In light of this, the World Health Organization (WHO) in 2004 created Asian-specific BMI trigger points for public health action. BMI cut-off points traditionally used for overweight and obesity (≥25 kg/m^2^ and ≥30 kg/m^2^, respectively) were lowered to ≥23 kg/m^2^ and ≥27.5 kg/m^2^ to represent ‘increased risk’ and ‘high-risk’ categories, respectively [24].

In order to improve public health screening and to develop ethnic-specific CVD prevention strategies in diverse communities, the relationship of obesity to cardiometabolic risk factors requires additional study. Therefore, the objective of this analysis is to compare the association between the World Health Organization’s Asian-specific BMI trigger points with self-reported cardiometabolic health amongst Asian-Canadian sub-groups.

## Methods

### Data source

This population-based analysis is based on data from six cycles of the Canadian Community Health Survey (CCHS; Cycles 1.1, 2.1, 3.1, 2007, 2008, and 2009), obtained through the limited data access program at the York University chapter of the Toronto Research Data Center of Statistics Canada.

The CCHS is a national cross-sectional survey that collects information related to health status, health care utilization and health determinants for the Canadian population [25]. It relies upon a large sample of respondents and is designed to provide reliable estimates at the health region level. Data collection occurred every two years prior to 2007 (i.e. cycles 1.1 (2001), 2.1 (2003) and 3.1 (2005)) and annually starting in 2007 (cycles 2007, 2008 and 2009). Interviews were conducted both in person and over the telephone. Three sampling frames were used to select the sample of households: 49% of the sample of households came from an area frame, 50% came from a list frame of telephone numbers and the remaining 1% came from a Random Digit Dialing (RDD) sampling frame.

The CCHS targets persons aged 12 years or older who are living in private dwellings in the ten provinces and the three territories. Excluded from this survey are persons living on aboriginal settlements, reserves, or crown lands, residents of institutions, full-time members of the Canadian Armed Forces and residents of certain remote regions. Its coverage is in the range of 98% in the provinces, but varies across other regions (Territories: 98%; Yukon: ∼90%; Northwest Territories: 97%, and; Nunavut: 71% (due to the exclusion of some remote regions)). To provide reliable estimates, a sample of 65 000 respondents is required on an annual basis for a total of approximately 130 000 respondents per every 2-year cycle.

Consistent with previous studies [18], [26], [27], the six survey cycles were pooled to obtain a sufficient sample size for the exploration of sub-group differences [28], [29].

### Study sample

After combining the six cycles of the CCHS, there were a total of 27 531 participants who reported being from one of the eight Asian ethnic groups. Following additional exclusions based on age <18 or ≥65 (n = 6 254), those who were pregnant at time of interview (n = 330), those missing BMI measurements (n = 619), those in the top 1% of BMI (i.e. BMI>35.7, n = 203) and those who were underweight (i.e. BMI<18.5, n = 1 331), the final analytic sample included 18 794 survey participants. The survey population was then weighted to be representative of the Canadian population between the survey years (2001–2009) [29], [30].

### Study variables

#### Independent (exposure) variables

All participants were asked to self-ascribe which cultural and racial background they were from. To assess Asian ancestry, only participants who self-ascribed an ethnicity as Chinese, South Asian (e.g., East Indian, Pakistani, Sri Lankan), Filipino, Southeast Asian (e.g., Cambodian, Indonesian, Laotian, Vietnamese), Arab, West Asian (e.g., Afghan, Iranian), Japanese, and Korean were retained for further analysis. The classification of Arabs as an Asian subgroup has been used in previous studies [18], [27], [31], [32]. Self-reported height without shoes (in metres) and weight (in kilograms) was used to place respondent’s into the WHO’s Asian specific trigger points for public health action representing ‘increased risk’ (BMI ≥23 kg/m^2^) and ‘high risk’ (BMI ≥27.5 kg/m^2^) [24].

#### Dependent (outcome) variables

Participants were asked about “long-term conditions” which were expected to last (or had already lasted) 6 months or more and that had been diagnosed by a health professional. Obesity-related cardiovascular conditions in the current analysis included self-reported high blood pressure (yes/no), diabetes (yes/no), heart disease (yes/no), and a composite variable of the presence of ‘at least one cardiometabolic condition’ (i.e. the presence of either high blood pressure, diabetes or heart disease).

#### Covariates

Demographic characteristics included sex, age of participant at time of survey, marital status (single/never married vs. other), highest level of education attained (less than secondary school graduation, secondary school graduation, some post-secondary, post-secondary graduation), household income, urban versus rural dwelling, immigrant status (non-immigrant vs. immigrant), length of time since immigration (years), and ability to ‘converse in English’ (yes/no). Income adequacy (i.e., lowest, lower-middle, upper-middle and highest income) was subsequently estimated using annual household income and household size as defined by Statistics Canada [33], [34].

Lifestyle characteristics included sedentary leisure time, leisure time physical activity, daily fruit and vegetable consumption, stress level, smoking status (“never smoked” vs “ever smoked”) and alcohol consumption (“did not drink in the last 12 months”, “occasional drinker”, and “regular drinker”). Sedentary leisure time (defined as the total number of hours per week respondents spent reading, watching television or videos, playing video games and on the computer) was categorized into 3 groups by tertiles (≤14 hrs/week, 15–24 hrs/week, and ≥25 hrs/week). A leisure time physical activity index (PAI; kcal/kg/day; kkd) was created; this variable reflects the average daily energy expenditure of leisure time activities in the past three months and is based on self-reported frequency and duration of physical activity along with the metabolic equivalent of each activity. Daily consumption of fruits and vegetables was quantified as the number of times (frequency) per day, rather than the amount consumed. Participants were asked to rate their self-perceived life stress on most days (“not at all stressful”, “not very stressful”, “a bit stressful”, “quite a bit stressful”, and “extremely stressful”).

### Statistical analysis

The combination of data from different cycles required a recalculation of sample weights to represent the characteristics of the pooled sample, which covers the combined time periods of the individual cycles. The original sampling weights were rescaled by a constant factor (α_i_ = 1/k, where k is equal to the number of cycles used), and the weighted proportions (%) of each variable was estimated. Statistical significance for continuous and categorical variables was assessed by ANOVA and χ^2^, respectively, for the overall sample and for each ethnic group. To check for effect modification by ethnicity, a general linear model (GLM) was used to test for interactions between BMI and ethnicity on cardiometabolic conditions. In all models (unadjusted, adjusted for demographics, and adjusted for demographics and lifestyle), there were significant interactions between ethnicity and BMI on all disease outcome measures (p<0.05).

Three logistic regression models were subsequently used to explore the independent and joint effects of ethnicity and overweight/obesity on cardiometabolic-related health. First, the odds of obesity-related chronic disease in Asian sub-groups (compared to South Asians; OR = 1.00) was estimated after accounting for various demographic, lifestyle, and acculturation characteristics. Second, logistic regression was used to estimate the odds of cardiometabolic diseases by BMI categories (compared to the ‘acceptable risk’ BMI category (≥18.5–23 kg/m^2^; OR = 1.00)) within each ethnic group. Finally, an overall analysis using South Asians in the ‘acceptable risk’ category as the referent group was conducted to examine the effect of obesity on chronic conditions across all BMI and Asian ethnic groups concurrently. Consistent with Statistics Canada guidelines, all cells with less than 10 observations, or a coefficient of variation ≥33% were suppressed [29]. All analyses were conducted using SAS version 9.2 (Cary, NC, U.S.A) with statistical significance was set at an alpha of 0.05.

## Results

### Descriptive characteristics

Characteristics of participants are presented in **Table 1**. The mean age of the pooled sample was 38.7 years, and 52.8% were male. Of the eight Asian ethnic groups, Chinese and South Asians accounted for the majority of the study sample (approximately 31% each), while Japanese accounted for the fewest (1.6%). The mean BMI overall was 24 kg^.^m^−2^, with a distribution of 43.4%, 41.5% and 15.1% in the ‘acceptable risk’, ‘increased risk’ and ‘high risk’ categories, respectively, based on WHO’s Asian-specific BMI trigger points. Eighty-five percent of the sample identified themselves as immigrants to Canada. Furthermore, the majority of respondents had a household education level of at least college or university, and belonged to the ‘Upper Middle’ or the ‘Highest’ income quartiles.

**Table 1:** Characteristics of overall study sample, Canada, 2001–2009.

Characteristic		Weighted Frequency	Percent %	*p*
Sex	Male	1 050 611	52.8	<0.001
	Female	940 441	47.2	
Self-reported Ethnicity	Chinese	628 857	31.6	<0.001
	South Asian	625 916	31.4	
	Filipino	231 719	11.6	
	Southeast Asian	156 683	7.9	
	Arab	141 199	7.1	
	West Asian	96 783	4.9	
	Japanese	32 682	1.6	
	Korean	77 213	3.9	
BMI Category (kg/m^2^) – Asian	18.5–<23	863 919	43.4	<0.001
	23–<27.5	825 751	41.5	
	≥27.5	301 383	15.1	
Marital Status	Single, Never Married	549 683	27.6	<0.001
	Everyone else	1 441 370	72.4	
Highest Household Education Level	Less than high schoolgraduation	58 448	3.2	<0.001
	High school graduation	173 816	9.4	
	Some post-secondary	102 668	5.6	
	College or university degree	1 515 356	81.9	
Household Income Quartile	Lowest income quartile	222 356	12.9	<0.001
	Lower middle income quartile	366 876	21.3	
	Upper middle income quartile	545 034	31.7	
	Highest income quartile	586 735	34.1	
Can have a conversationin English		1 792 143	90.0	<0.001
Immigrant to Canada		1 678 269	85.0	<0.001
Smoking	Never Smoked	1 300 063	65.3	<0.001
	Ever Smoked	690 990	34.7	
Alcohol	Regular	783 389	39.5	<0.001
	Occasional Drinker	386 550	19.5	
	Did not drink in last year	814 260	41.0	
Physician diagnosed highblood pressure		192 267	9.7	<0.001
Physician diagnosed diabetes		82 440	4.1	<0.001
Physician diagnosed heartdisease		34 296	1.7	<0.001
At least 1 chronic disease		260 789	13.1	<0.001
Physical Activity Level	Active	376 597	19.4	<0.001
	Moderate	403 684	20.8	
	Inactive	1 163 972	59.9	
Sedentary time (Tertiles)	< = 14 hrs/wk	396 700	36.5	0.004
	15–24 hrs/wk	364 421	33.5	
	25−>45 hrs/wk	327 215	30.1	
Urban/Rural	Urban	1 950 562	98.0	<0.001
	Rural	40 490	2.0	
Stress	Not at all stressful	200 860	10.1	<0.001
	Not very stressful	441 059	22.2	
	A bit stressful	872 218	44.0	
	Quite a bit stressful	395 858	20.0	
	Extremely stressful	73 549	3.7	
Daily Energy Expenditure –kcal/kg/day (mean, 95% CI)		1.7 (1.54–1.88)		
Age – years (mean, 95% CI)		38.7 (38.38–39.03)		
Body Mass Index – kg/m^2^(mean, 95% CI)		24.0 (23.73–24.24)		
Frequency of Daily Fruits/Vegetable Consumption(mean, 95% CI)		4.7 (4.52–4.82)		
Household size		3.7 (3.53–3.85)		

**Table 2** presents the full descriptive characteristics of the sample by Asian sub-groups. Among Asian sub-groups, Arabs (29.2%), West Asians (19.5%), and South Asians (19.3%) had the highest prevalence of individuals in the ‘high risk’ BMI category, while Chinese (8.2%) had the lowest. The Chinese sub-group also had the highest percentage of respondents in the ‘acceptable risk’ BMI category (56.2%). Similarly, mean BMI was highest among Arabs (25.5 kg^.^m^−2^) and lowest in Chinese (23 kg^.^m^−2^). While Japanese and Koreans were most likely to report having ever smoked cigarettes (50.8% and 49.8%, respectively) and being regular drinkers of alcohol (60.7% and 58.5%, respectively), they were also most likely to be classified as physically active (23% and 26.7%, respectively).

**Table 2:** Characteristics of Asian sub-groups, Canada, 2001–2009.

		Chinese (%)	South Asian (%)	Filipino (%)	Southeast Asian (%)	Arab (%)	West Asian (%)	Japanese (%)	Korean (%)
	Weighted Frequency	n = 628 857	n = 625 916	n = 231 720	n = 156 683	n = 141 199	n = 96 783	n = 32 682	n = 77 213
Sex	Male	52.9	54.3	44.7	54.9	58.7	55.4	43.5	48.6
	Female	47.1	45.7	55.3	45.1	41.3	44.6	56.5	51.5
BMI Category(kg/m^2^) - Asian	18.5–<23	56.2	34.5	40.3	44.1	30.2	37.7	49.4	47.9
	23–<27.5	35.6	46.2	44.8	42.7	40.6	42.8	38.1	39.9
	≥27.5	8.2	19.3	14.9	13.2	29.2	19.5	12.5	12.2
MaritalStatus	Single, Never Married	29.2	23.6	30.5	26.8	31.3	29.2	27.3	31.9
	Everyone else	70.8	76.4	69.5	73.2	68.7	70.9	72.7	68.1
HighestHouseholdEducationLevel	Less than high school	2.8	3.5	0.5	6.1	3.7	6.6	1.0	0.7
	High school graduation	10.2	10.8	3.3	13.5	5.2	11.1	5.9	8.0
	Some postsecondary	6.6	5.2	3.4	6.4	5.6	4.6	4.6	6.3
	College or university	80.5	80.5	92.7	73.9	85.5	77.7	88.5	85.0
Household Income Quartile	Lowest	13.4	11.5	7.8	11.6	22.5	21.9	9.4	12.9
	Lower middle	19.2	23.9	19.7	20.5	26.5	19.5	13.6	20.2
	Upper middle	30.6	30.9	35.9	36.2	29.8	27.6	22.2	37.6
	Highest	36.9	33.7	36.7	31.6	21.2	31.0	54.8	29.4
Can have aconversationin English		84.5	93.4	97.4	88.2	84.7	93.7	97.3	91.7
Immigrant toCanada		83.5	85.5	88.9	86.4	87.6	95.2	43.1	78.7
Smoking	Never Smoked	67.1	71.9	63.2	62.9	52.4	56.0	49.2	50.2
	Ever Smoked	32.9	28.1	36.8	37.1	47.6	44.0	50.8	49.8
Alcohol	Regular	39.7	35.9	37.1	45.6	34.1	42.4	60.7	58.5
	Occasional Drinker	25.8	12.2	28.8	19.6	11.2	15.8	16.9	19.6
	Did not drink in last year	34.4	51.9	34.2	34.8	54.7	41.8	22.4	21.9
Physiciandiagnosedhigh bloodpressure		8.5	9.8	15.4	9.8	8.0	4.7	10.2	9.6
Physiciandiagnoseddiabetes		2.3	6.2	4.5	3.8	2.3	6.0	3.4	2.9
Physiciandiagnosedheart disease		1.8	1.7	1.0	0.3	1.8	6.5	1.9	0.8
At least one cardiometaboliccondition		11.1	14.7	18.4	12.3	9.2	11.7	11.7	11.8
Physical Activity Level	Active	17.3	19.7	20.4	20.8	17.3	21.9	23.3	26.7
	Moderate	21.1	21.1	21.0	18.7	20.4	18.8	29.0	19.0
	Inactive	61.6	59.3	58.6	60.5	62.3	59.3	47.8	54.4
Sedentary time (Tertiles)	< = 14 hrs/wk	28.7	44.5	40.2	43.2	32.9	34.6	28.9	23.9
	15–24 hrs/wk	34.5	30.2	33.9	31.7	36.7	34.4	37.2	44.5
	25−>45 hrs/wk	36.8	25.3	25.9	25.1	30.4	31.0	33.9	31.7
Urban/Rural	Urban	98.7	97.5	98.1	97.3	98.7	98.6	96.6	95.5
	Rural	1.4	2.5	1.9	2.7	1.3	1.4	3.4	4.5
Stress	Not at all stressful	8.9	12.1	11.2	11.0	8.4	7.5	8.8	6.2
	Not very stressful	23.8	20.5	26.3	20.7	15.5	15.5	33.3	30.6
	A bit stressful	45.6	43.0	42.2	47.4	41.7	43.5	41.5	43.4
	Quite a bit stressful	18.9	20.1	17.5	17.9	28.0	27.8	11.4	17.6
	Extremely stressful	2.7	4.4	2.9	3.1	6.3	5.8	5.0	2.2
Daily EnergyExpenditure –kcal/kg/day(mean, 95% CI)		1.56 (1.43–1.7)	1.72 (1.51–1.93)	1.92 (1.64–2.2)	1.76 (1.52–2)	1.67 (1.36–1.98)	1.73 (1.41–2.05)	1.89 (1.6–2.19)	2 (1.8–2.21)
Age – years(mean, 95% CI)		39.72 (38.84–40.59)	38.04 (37.52–8.56)	39.24 (38.51–39.97)	38.16 (37.2–39.12)	36.7 (35.63–37.76)	37.94 (35.97–39.91)	43.03 (40.78–45.27)	38.07 (36.73–39.41)
Body Mass Index– kg/m^2^ (mean,95% CI)		23 (22.83–23.18)	24.62 (24.4–24.85)	24.15 (23.92–24.39)	23.76 (23.52–24)	25.5 (25.17–25.83)	24.63 (24.22–25.05)	23.4 (23.03–23.77)	23.44 (22.9–23.98)
Frequency of Daily Fruits/Vegetable Consumption(mean, 95% CI)		4.35 (4.18–4.52)	4.85 (4.74–4.97)	4.76 (4.55–4.97)	4.63 (4.37–4.89)	4.82 (4.57–5.07)	4.99 (4.36–5.62)	4.7 (4.44–4.96)	4.87 (4.56–5.17)
Household size		3.33 (3.22–3.43)	4.07 (3.86–4.28)	3.86 (3.75–3.97	3.82 (3.65–3.99)	3.70 (3.29–4.10)	3.51 (3.23–3.78)	2.94 (2.67–3.21)	3.25 (3.08–3.42)

**Figure 1:**
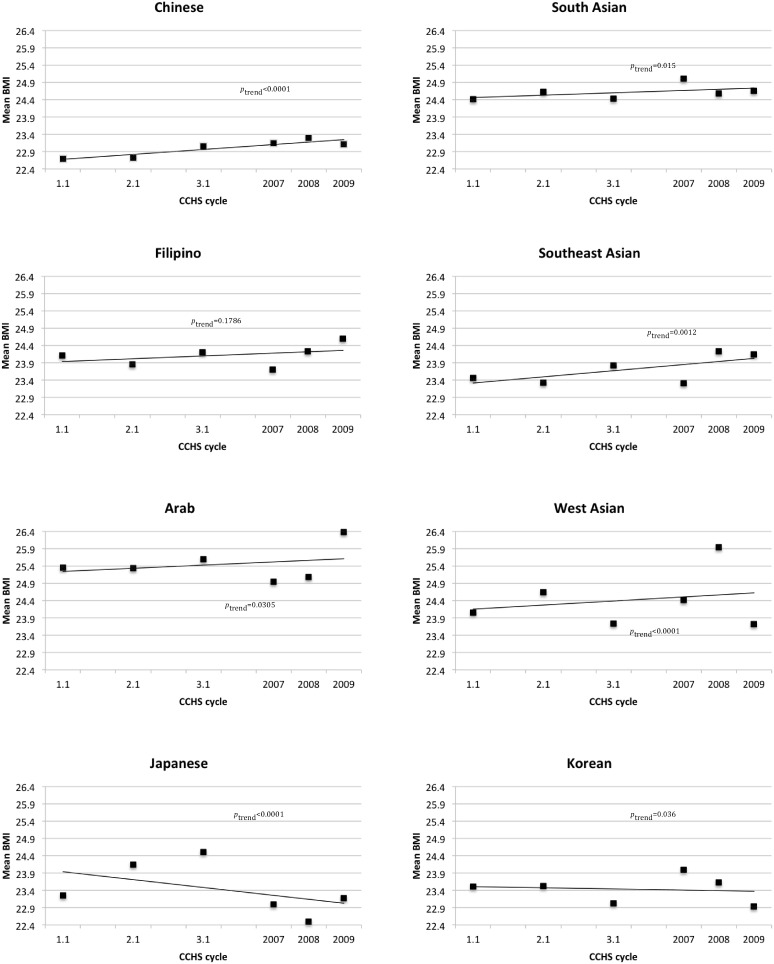
Weighted-unadjusted mean BMI across CCHS cycles (2001–09) by Asian sub-groups.

Mean BMI over the 6 CCHS cycles (9 year period) has increased significantly for each Asian ethnicity except Filipino, Japanese and Korean. This trend was strongest in Southeast Asians and Chinese (**Figure 1**). There was no significant difference in mean BMI for Filipinos from 2001 to 2009 (p_trend_ = 0.18), whereas mean BMI decreased in Japanese and Korean subgroups (p_trend_ <0.05).

**Figure 2:**
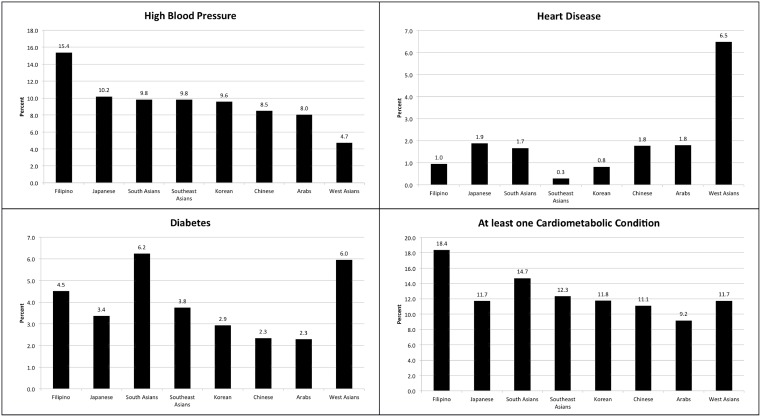
Prevalence of cardiometabolic conditions by Asian sub-groups, 2001–09.

**Figure 2** presents the prevalence of self-reported cardiovascular conditions according to ethnicity. In general, Filipinos were most likely to report having high blood pressure (15.4%) and ‘at least one cardiometabolic condition’ (18.4%), whereas South Asians (6.2%) and West Asians (6.0%) were most likely to report having diabetes. West Asians also reported the highest prevalence of heart disease (6.5%).

### Association between ethnicity and cardiometabolic conditions

The odds of reporting a physician-diagnosed cardiometabolic condition (i.e. high blood pressure, diabetes, heart disease, or ‘at least one cardiometabolic condition’) for each ethnic group compared to South Asians (OR = 1.00) is presented in **Table 3**. After adjusting for covariates, when compared to South Asians, Filipinos reported a 60% greater likelihood of high blood pressure *(OR, lower CI-upper CI)* (1.6, 1.05–2.44). As expected, nearly all Asian sub-groups had lower odds of diabetes compared to South Asians. Compared to South Asians, the odd of having ‘at least one cardiometabolic condition’ was significantly lower in Chinese (0.63, 0.44–0.90) and Arabs (0.38, 0.28–0.51), but significantly higher in Filipinos (1.29, 1.04–1.62); no differences were observed in the other Asian ethnic groups.

**Table 3:** Multivariable-adjusted odds ratios of ‘at least one cardiometabolic condition’ for Asian sub-groups compared to South Asians.

	High Blood Pressure		Diabetes		Heart Disease		At least one Cardiometabolic Condition	
Self-reported Ethnicity	OR* (95% CI)							
South Asian	***1.00 (referent)***		***1.00 (referent)***		***1.00 (referent)***		***1.00 (referent)***	
Chinese	0.76	(0.55–1.06)	**0.25**	**(0.12–0.5)**	1.52	(0.47–4.9)	**0.63**	**(0.44–0.9)**
Filipino	**1.6**	**(1.05–2.44)**	**0.61**	**(0.39–0.97)**	0.92	(0.33–2.54)	**1.29**	**(1.04–1.62)**
Southeast Asian	1.09	(0.64–1.85)	**0.31**	**(0.15–0.67)**	**0.22**	**(0.05–0.89)**	0.72	(0.37–1.4)
Arab	**0.68**	**(0.51–0.91)**	**0.15**	**(0.07–0.3)**	1.24	(0.57–2.71)	**0.38**	**(0.28–0.51)**
West Asian	**0.31**	**(0.14–0.71)**	0.66	(0.4–1.1)	**3.86**	**(2.4–6.22)**	0.75	(0.47–1.21)
Japanese	0.53	(0.13–2.2)	**0.09**	**(0.02–0.45)**	1.51	(0.29–7.74)	0.46	(0.14–1.53)
Korean	1.03	(0.49–2.16)	0.50	(0.23–1.1)	1.06	(0.32–3.49)	0.87	(0.48–1.56)

*Adjusted for BMI, demographic (age, sex, marital status, stress level, smoking, alcohol, household education level, household income quartile, English proficiency, immigrant status, age at immigration, length of time since immigration, urban/rural), and lifestyle (sedentary time, daily energy expenditure, daily fruit and vegetable consumption) variables.

### Association between ethnicity, BMI category and ‘at least one cardiometabolic condition’

**Table 4** shows the adjusted odds of ‘at least one cardiometabolic condition’ for individuals in the ‘increased risk’ and ‘high risk’ BMI categories compared to those in the ‘acceptable risk’ BMI category in each ethnic sub-group. Overall, Asians in the ‘increased’ and ‘high’ risk categories were two- and four- times more likely to report ‘at least one cardiometabolic condition’ compared to those in the ‘acceptable risk’ category. However, this effect was not consistent across sub-groups. Specifically, odds were greatest for Chinese (increased: 3.6, 2.34–5.63; high: 8.9, 3.6–22.01), lower for South Asian (increased: 1.74, 1.23–2.46; high: 3.37, 2.02–5.65), and only reaching statistical significance in the ‘high’ risk category for Filipinos (2.39, 1.27–4.47), Southeast Asians (3.38, 1.4–8.16) and Koreans (3.15, 1.36–7.33).

**Table 4:** Association between BMI and having ‘at least one cardiometabolic condition’.

		At least 1 CardiometabolicCondition(referent = normal-weightBMI category in eachethnic group)		At least oneCardiometabolicCondition(referent = normal-weight South Asians)			
Self-reported Ethnicity	BMICategory	OR* (95% CI)		OR* (95% Confidence Interval)			
Chinese	18.5–<23	*1.00 (referent)*		0.41		(0.24–0.69)	
	23–<27.5	3.63	(2.34–5.63)		1.27		(0.9–1.81)
	≥27.5	8.9	(3.6–22.01)		3.05		(1.21–7.67)
South Asian	18.5–<23	*1.00 (referent)*		*1.00 (referent)*			
	23–<27.5	1.74	(1.23–2.46)		1.79		(1.18–2.71)
	≥27.5	3.37	(2.02–5.65)		3.55		(2.26–5.59)
Filipino	18.5–<23	*1.00 (referent)*		1.33		(0.76–2.33)	
	23–<27.5	2.12	(0.98–4.58)		2.59		(1.5–4.46)
	≥27.5	2.39	(1.27–4.47)		3.26		(2.18–4.89)
Southeast Asian	18.5–<23	*1.00 (referent)*		1.02		(0.5–2.09)	
	23–<27.5	0.87	(0.51–1.5)		0.9		(0.42–1.9)
	≥27.5	3.38	(1.4–8.16)		2.5		(0.56–11.16)
Arab	18.5–<23	*1.00 (referent)*		0.67		(0.25–1.85)	
	23–<27.5	0.97	(0.34–2.79)		0.58		(0.37–0.91)
	≥27.5	2.32	(0.97–5.55)		1.5		(0.88–2.55)
West Asian	18.5–<23	*1.00 (referent)*		0.96		(0.28–3.31)	
	23–<27.5	1.21	(0.16–9.08)		1.47		(0.67–3.24)
	≥27.5	1.32	(0.15–11.67)		2.75		(1.26–6.01)
Japanese	18.5–<23	*1.00 (referent)*		0.53		(0.13–2.18)	
	23–<27.5	0.86	(0.13–5.79)		0.49		(0.16–1.52)
	≥27.5	1.72	(0.06–48.39)		2.15		(0.18–25.95)
Korean	18.5–<23	*1.00 (referent)*		0.84		(0.39–1.77)	
	23–<27.5	2.04	(0.9–4.62)		1.49		(0.79–2.83)
	≥27.5	3.15	(1.36–7.33)		3.17		(1.04–9.67)
Asians Overall	18.5–<23	*1.00 (referent)*					
	23–<27.5	2.09	(1.55–2.82)				
	≥27.5	4.16	(3–5.78)				

*Adjusted for demographic (age, sex, marital status, stress level, smoking, alcohol, household education level, household income quartile, English proficiency, immigrant status, age at immigration, length of time since immigration, urban/rural), and lifestyle (sedentary time, daily energy expenditure, daily fruit and vegetable consumption) variables.

Finally, we calculated the adjusted OR of ‘at least one cardiometabolic condition’ for each BMI category in each Asian sub-group compared to South Asians in the ‘acceptable risk’ weight category (**Table 4**). South Asians (increased: 1.79, 1.18–2.71; high: 3.55, 2.26–5.59) and Filipinos (increased: 2.59, 1.5–4.46; high: 3.26, 2.18–4.89) had significantly higher odds of ‘at least one cardiometabolic condition’ than South Asians in the ‘acceptable risk’ weight category. Among the other ethnic sub-groups, only the ‘high risk’ weight category for Chinese (3.05, 1.21–7.67), West Asians (2.75, 1.26–6.01), and Koreans (3.17, 1.04–9.67) were at higher odds versus South Asians in the ‘acceptable risk’ category. Finally, Chinese in the ‘acceptable risk’ category had significantly lower odds of having ‘at least one cardiometabolic condition’ than normal weight South Asians (0.41, 0.24–0.69).

## Discussion

While Asians are often studied as a broad group, the results of this study provide further evidence of the heterogeneity in the obesity-health relationship across Asian ethnicities. When compared to a common referent group (i.e. South Asians in the ‘acceptable risk’ weight category), the relationship between excess weight and poor cardiometabolic health is strongest in the Filipino and South Asian sub-groups. Within sub-groups, the association between ‘increased’ and ‘high’ risk BMI categories and ‘at least one cardiometabolic condition’ also varies and is highest among Chinese.

### Asians are different from each other in their BMI and CVD risks

These analyses confirm previous findings that the classification of Asians as a homogenous group can mask health risk amongst diverse Asian populations [19], [20], [35]. Specifically, these results demonstrate that Asian sub-groups differed from each other in cardiovascular risk factors such as smoking, physical activity level, alcohol, BMI, high blood pressure, diabetes and heart disease. However, in fully adjusted models, only Filipinos had higher odds of ‘at least one cardiometabolic condition’ compared to South Asians, whereas Chinese and Arabs had lower odds, and no difference was observed in the other ethnic sub-groups. All Asian sub-groups had lower odds of diabetes compared to South Asians, except for West Asians and Koreans (where no difference was observed).

Two notable Canadian studies have examined ethnic differences in obesity and cardiovascular disease that corroborate our findings [16], [18]. In the first, Chiu et al. (2010) examined cardiovascular risk among people living in Ontario, Canada (between 1996 and 2007) who self-ascribed their ethnicity as White, South Asian, Chinese or black [16]. They found considerable variations by ethnicity in the prevalence of smoking (South Asian: 8.6%, Chinese: 8.7%, black: 11.4% and White: 24.8%), obesity (Chinese: 2.5%, South Asian: 8.1%, black: 14.1%, and White: 14.8%), diabetes mellitus (White: 4.2%, Chinese: 4.3%, South Asian: 8.1%, and black: 8.5%) and hypertension (White: 13.7%, Chinese: 15.1%, South Asian: 17%, and black: 19.8%). Age- and sex- standardized mean BMI was lowest among the Chinese respondents (22.3 kg.m^−2^), followed by South Asian (24.2 kg.m^−2^), White (25.3 kg.m^−2^) and black (25.5 kg.m^−2^) populations. Overall, Chinese respondents had the most favourable cardiovascular risk factor profile, with 4.3% of the population reporting two or more major cardiovascular risk factors, followed by the South Asian (7.9%), White (10.1%) and black (11.1%) respondents.

In the second study, Liu et al. pooled data from three cycles (2000, 2003 and 2005) of the CCHS to examine the prevalence of CVD and associated risk factors in the various Canadian ethnic groups [18]. The prevalence of cardiovascular risk factors in this study is consistent with the Liu paper. Compared to White individuals, people from most visible minorities were less likely to smoke, more likely to be physically inactive, and were less likely to be obese. After adjustment for socio-demographic characteristics and chronic conditions, Liu et al. found that diabetes and hypertension were significantly more prevalent amongst South Asians (adjusted OR 2.17 for diabetes and 1.18 for hypertension), Filipino or South-East Asian respondents (adjusted OR 1.58 for diabetes and 1.54 for hypertension) than White respondents. No differences were seen for other Asian ethnicities as compared to Whites.

### Ethnic variation in health risk associated with obesity

Building on the work of others [16], [18], this study examines the relationship between obesity and cardiometabolic risk among Asian sub-groups in Canada. In the present study, when all Asian sub-groups were collapsed, those within the ‘increased’ or ‘high’ BMI categories were 2- to 4- times more likely to have ‘at least one cardiometabolic condition’. When analyses were repeated within each Asian ethnic group, differences emerged. Most strikingly, the relationship between BMI and ‘at least one cardiometabolic condition’ was strongest in Chinese, resulting in 3.6 and 9 times greater odds in the ‘increased’ and ‘high’ risk BMI categories, respectively.

Our results are consistent with other literature on the effect of obesity on cardiovascular health risk in Asian populations [36]–[46]. Several studies have shown that the association between BMI and cardiometabolic risks is steepest in Chinese compared to other ethnicities [38], [41], [46]. For example, Katz et al. showed that the adjusted incidence difference for hypertension per 1000 persons in young adults with a BMI of 25 vs. those of BMI of 21 was 83 for Chinese, 50 for Blacks and 30 for Whites. Amongst middle-aged adults, similar patterns are seen, with incidence differences of 137 for Chinese, 49 for Blacks, and 54 for Whites [41].

There are several possible explanations for the observed differences in the relationship between obesity and cardiometabolic risk factors between Asian ethnic sub-groups. Both environmental and genetic factors are likely to be important in determining CVD risk. Lifestyle changes and increasing affluence have led to a high prevalence of obesity, insulin resistance, T2DM and CVD among Asians living in the West. However, previous research indicates that differences in CVD cannot be explained by differences in conventional cardiovascular risk factors alone [47]. Having adjusted for these modifiable/behavioural characteristics in this study, other factors such as differences in the relationship between BMI, BF%, and health are potential contributors to the observed differences.

Differences in the association between excess weight and cardiometabolic health risks among Asian sub-groups may is attributed in part to differences in BMI-Fat Mass (FM)% as a result of differences in body build and/or frame size. BMI does not distinguish between individuals or populations who have very long or short legs relative to torso length, and BMI will tend to underestimate obesity amongst those with long legs and over estimate obesity among those with short legs relative to torso length [3], [48], [49]. It is well known that ethnic groups differ in frame size and in relative leg length (relative sitting height) and that his has an impact on BMI [3], [11], [48], [50]–[52]. The distribution of body fat is also different in Asians compared with Europeans whereby Asians show a greater proportion of visceral abdominal tissue (VAT) for a given total body fat [53]–[61]. In turn, VAT has been shown to be an independent risk factor for CHD, hypertension, T2DM and impaired glucose tolerance [4], [51], [53], [55], [62]–[64].

Several recent studies provide further insight into differences in body composition and health risk in Asian sub-groups. In the Multicultural Community Health Assessment Trial (M-CHAT) [13], [65], while BMI and WC were highly correlated with total and regional measures of adiposity in each ethnic group, at any BMI, Chinese participants had a similar FM% to that of Europeans, whereas South Asians had 3.9% more. Above a WC of 71 cm, Chinese participants had considerably more VAT than European-Canadians, whereas South Asians had significantly more VAT than Europeans at all but the most extreme WC category (>105 cm) [13], [65].

### Strengths and limitations

Notable strengths of the current analysis include the use of a large, nationally representative sample, disaggregated into each Asian sub-group, and the inclusion of important socio-demographic and lifestyle variables associated with obesity and cardiovascular risk factors. Unlike previous studies that have used the ‘White’ population as the referent group, the current analysis opted for an internal comparison group within the broader ‘Asian’ categorization, as even among persons classified as “White”, there is considerable variation in factors such as country of origin, birth cohort, and acculturation that may confound the relationship between obesity and health risk [37], [66]. By using the more sensitive WHO cut-points for BMI in Asians, this analysis is able to capture variations in health risk that might otherwise have been missed.

There are also several limitations that need to be noted. First, given that the CCHS relies on self-reported data, there is potential for both recall and healthy responder bias. For example, the possibility of under-reporting BMI (via an underestimation of weight among females and an overestimation of height amongst males) cannot be excluded [67]. Reporting of obesity may also vary by ethnicity, along with differences in the way people experience and label diseases, symptoms, and various lifestyle-related behaviours. The underestimation of chronic conditions by participants is also a possibility, but would have biased our results towards the null. The limitations of using BMI cut-offs are also well known [49], [51], [68], [69]; however, due to the relative ease of use [70] and high specificity and validity [71], [72], BMI may be considered a reasonable proxy of weight-related health risk in *population-based* studies [73], [74]. Furthermore, type of diabetes was not differentiated. Finally, despite the large overall sample size used in this study, analyses for heart disease within obese categories of some ethnic groups had to be suppressed, whereas others may be underpowered to detect a difference.

### Conclusion

Results of this study provide additional insight into the relationship between obesity and cardiovascular health across Asian sub-groups, as the appropriate classification of sub-populations is necessary if the mechanisms underlying such differences in health risk are to be understood and monitored. When taken together, higher odds of CVD associated with overweight and obesity for the Chinese, Filipino and South Asian groups, and the steeper association between excess adiposity and cardiovascular risk in Chinese, has important public health implications for targeted screening and culturally-specific interventions focusing on susceptible Asian-ethnic communities.

## References

[1] Statistics Canada (2011) Immigration and Ethnocultural Diversity in Canada. National Household Survey (NHS). Statistics Canada. Available: http://www12.statcan.gc.ca/nhs-enm/2011/as-sa/99-010-x/99-010-x2011001-eng.cfm. Accessed 2014 March 11.

[2] Statistics Canada (2010) Study: Projections of the diversity of the Canadian population. Available: http://www.statcan.gc.ca/daily-quotidien/100309/dq100309a-eng.htm. Accessed 2013 April 24.

[3] Deurenberg P, Deurenberg-Yap M (2003) Validity of body composition methods across ethnic population groups. Forum Nutr 56: 299–301. 15806909

[4] Forouhi NG, Sattar N (2006) CVD risk factors and ethnicity–a homogeneous relationship? Atheroscler Suppl 7: 11–19. 10.1016/j.atherosclerosissup.2006.01.003 16500156

[5] Kanaya AM, Adler N, Moffet HH, Liu J, Schillinger D, et al (2011) Heterogeneity of diabetes outcomes among asians and pacific islanders in the US: the diabetes study of northern California (DISTANCE). Diabetes Care 34: 930–937. 21350114 10.2337/dc10-1964 PMC3064053

[6] Lee JW, Brancati FL, Yeh HC (2011) Trends in the prevalence of type 2 diabetes in Asians versus whites: results from the United States National Health Interview Survey, 1997–2008. Diabetes Care 34: 353–357. 21216863 10.2337/dc10-0746 PMC3024348

[7] McNeely MJ, Boyko EJ (2004) Type 2 diabetes prevalence in Asian Americans: results of a national health survey. Diabetes Care 27: 66–69. 14693968 10.2337/diacare.27.1.66

[8] Oza-Frank R, Ali MK, Vaccarino V, Narayan KM (2009) Asian Americans: diabetes prevalence across U.S. and World Health Organization weight classifications. Diabetes Care 32: 1644–1646. 19509010 10.2337/dc09-0573 PMC2732150

[9] Palaniappan LP, Araneta MR, Assimes TL, Barrett-Connor EL, Carnethon MR, et al (2010) Call to action: cardiovascular disease in Asian Americans: a science advisory from the American Heart Association. Circulation 122: 1242–1252. 20733105 10.1161/CIR.0b013e3181f22af4 PMC4725601

[10] Anand SS, Yusuf S, Vuksan V, Devanesen S, Teo KK, et al (2000) Differences in risk factors, atherosclerosis, and cardiovascular disease between ethnic groups in Canada: the Study of Health Assessment and Risk in Ethnic groups (SHARE). Lancet 356: 279–284. 11071182 10.1016/s0140-6736(00)02502-2

[11] Deurenberg P, Deurenberg-Yap M, Guricci S (2002) Asians are different from Caucasians and from each other in their body mass index/body fat per cent relationship. Obes Rev 3: 141–146. 12164465 10.1046/j.1467-789x.2002.00065.x

[12] Deurenberg-Yap M, Deurenberg P (2003) Is a re-evaluation of WHO body mass index cut-off values needed? The case of Asians in Singapore. Nutr Rev 61: S80–87. 12828197 10.1301/nr.2003.may.S80-S87

[13] Lear SA, Humphries KH, Kohli S, Birmingham CL (2007) The use of BMI and waist circumference as surrogates of body fat differs by ethnicity. Obesity (Silver Spring) 15: 2817–2824. 18070773 10.1038/oby.2007.334

[14] Wulan SN, Westerterp KR, Plasqui G (2010) Ethnic differences in body composition and the associated metabolic profile: a comparative study between Asians and Caucasians. Maturitas 65: 315–319. 20079586 10.1016/j.maturitas.2009.12.012

[15] Barnes PM, Adams PF, Powell-Griner E (2008) Health characteristics of the Asian adult population: United States, 2004–2006. Adv Data: 1–22. 18271366

[16] Chiu M, Austin PC, Manuel DG, Tu JV (2010) Comparison of cardiovascular risk profiles among ethnic groups using population health surveys between 1996 and 2007. Cmaj 182: 774–780. 10.1503/cmaj.091676 PMC2871219 20403888

[17] Gholap N, Davies M, Patel K, Sattar N, Khunti K (2011) Type 2 diabetes and cardiovascular disease in South Asians. Prim Care Diabetes 5: 45–56. 20869934 10.1016/j.pcd.2010.08.002

[18] Liu R, So L, Mohan S, Khan N, King K, et al (2010) Cardiovascular risk factors in ethnic populations within Canada: results from national cross-sectional surveys. Open Med 4: e143–153. 21687334 PMC3090103

[19] Narayan KM, Aviles-Santa L, Oza-Frank R, Pandey M, Curb JD, et al (2010) Report of a National Heart, Lung, And Blood Institute Workshop: heterogeneity in cardiometabolic risk in Asian Americans In the U.S. Opportunities for research. J Am Coll Cardiol 55: 966–973. 20202512 10.1016/j.jacc.2009.07.075

[20] Palaniappan L, Wang Y, Fortmann SP (2004) Coronary heart disease mortality for six ethnic groups in California, 1990–2000. Ann Epidemiol 14: 499–506. 15310526 10.1016/j.annepidem.2003.12.001

[21] Araneta MR, Wingard DL, Barrett-Connor E (2002) Type 2 diabetes and metabolic syndrome in Filipina-American women: a high-risk nonobese population. Diabetes Care 25: 494–499. 11874936 10.2337/diacare.25.3.494

[22] Grandinetti A, Chang HK, Theriault A, Mor J (2005) Metabolic syndrome in a multiethnic population in rural Hawaii. Ethn Dis 15: 233–237. 15825969

[23] Palaniappan LP, Kwan AC, Abbasi F, Lamendola C, McLaughlin TL, et al (2007) Lipoprotein abnormalities are associated with insulin resistance in South Asian Indian women. Metabolism 56: 899–904. 17570249 10.1016/j.metabol.2007.01.020

[24] WHO Expert Consultation (2004) Appropriate body-mass index for Asian populations and its implications for policy and intervention strategies. Lancet 363: 157–163. 14726171 10.1016/S0140-6736(03)15268-3

[25] Statistics Canada (2007) Canadian Community Health Survey (CCHS). Available: http://www23.statcan.gc.ca/imdb/p2SV.pl?Function=getSurvey&SurvId=1630&InstaId=3359&SDDS=3226. Accessed 2014 August 8.

[26] Dogra S, Meisner BA, Ardern CI (2010) Variation in mode of physical activity by ethnicity and time since immigration: a cross-sectional analysis. Int J Behav Nutr Phys Act 7: 75. 20946636 10.1186/1479-5868-7-75 PMC2978119

[27] Tremblay MS, Perez CE, Ardern CI, Bryan SN, Katzmarzyk PT (2005) Obesity, overweight and ethnicity. Health Rep 16: 23–34. 16190322

[28] Statistics Canada. (2013) Other reference periods - Canadian Community Health Survey - Annual Component (CCHS). Available: http://www23.statcan.gc.ca/imdb/p2SV.pl?Function=getInstanceList&SurvId=3226&SurvVer=1&InstaId=15282&SDDS=3226&lang=en&db=imdb&adm=8&dis=2. Accessed 2014 March 11.

[29] Thomas S, Wannell B (2009) Combining cycles of the Canadian Community Health Survey. Health Rep 20: 53–58. 19388369

[30] Sarafin C, Simard M, Thomas S (2007) A Review of the Weighting Strategy for the Canadian Community Health Survey. SSC Annual Meeting, Proceedings of the Survey Methods Section.

[31] Brenner DR, Arora P, Garcia-Bailo B, Wolever TM, Morrison H, et al (2011) Plasma vitamin D levels and risk of metabolic syndrome in Canadians. Clin Invest Med 34: E377. 22129928 10.25011/cim.v34i6.15899

[32] Hou F, Picot G (2004) Visible minority neighbourhoods in Toronto, Montréal, and Vancouver. Canadian Social Trends 72: 8–13.

[33] Statistics Canada. Canadian Community Health Survey (CCHS) Cycle 1.1: Derived Variable (DV) Specifications.

[34] Statistics Canada. (2009) Canadian Community Health Survey (CCHS), 2008 (Annual component) and 2007–2008. Derived Variable (DV) Specifications. Master and share files. Available: http://www23.statcan.gc.ca/imdb-bmdi/pub/document/3226_D2_T9_V6-eng.pdf. Accessed 2014 March 22.

[35] Srinivasan S, Guillermo T (2000) Toward improved health: disaggregating Asian American and Native Hawaiian/Pacific Islander data. Am J Public Health 90: 1731–1734. 11076241 10.2105/ajph.90.11.1731 PMC1446402

[36] Chen Y, Copeland WK, Vedanthan R, Grant E, Lee JE, et al (2013) Association between body mass index and cardiovascular disease mortality in east Asians and south Asians: pooled analysis of prospective data from the Asia Cohort Consortium. Bmj 347: f5446. 24473060 10.1136/bmj.f5446 PMC3788174

[37] Chiu M, Austin PC, Manuel DG, Shah BR, Tu JV (2011) Deriving ethnic-specific BMI cutoff points for assessing diabetes risk. Diabetes Care 34: 1741–1748. 21680722 10.2337/dc10-2300 PMC3142051

[38] Colin Bell A, Adair LS, Popkin BM (2002) Ethnic differences in the association between body mass index and hypertension. Am J Epidemiol 155: 346–353. 11836199 10.1093/aje/155.4.346

[39] Davis J, Juarez D, Hodges K (2013) Relationship of ethnicity and body mass index with the development of hypertension and hyperlipidemia. Ethn Dis 23: 65–70. 23495624 PMC3726536

[40] Foulds HJ, Bredin SS, Warburton DE (2012) The relationship between hypertension and obesity across different ethnicities. J Hypertens 30: 359–367. 22236971 10.1097/HJH.0b013e32834f0b86

[41] Katz EG, Stevens J, Truesdale KP, Cai J, North KE, et al (2013) Associations of body mass index with incident hypertension in American white, American black and Chinese Asian adults in early and middle adulthood: the Coronary Artery Risk Development in Young Adults (CARDIA) study, the Atherosclerosis Risk in Communities (ARIC) study and the People's Republic of China (PRC) study. Asia Pac J Clin Nutr 22: 626–634. 24231024 10.6133/apjcn.2013.22.4.12 PMC4053207

[42] Low S, Chin MC, Ma S, Heng D, Deurenberg-Yap M (2009) Rationale for redefining obesity in Asians. Ann Acad Med Singapore 38: 66–69. 19221673

[43] Nguyen TT, Adair LS, Suchindran CM, He K, Popkin BM (2009) The association between body mass index and hypertension is different between East and Southeast Asians. Am J Clin Nutr 89: 1905–1912. 19369374 10.3945/ajcn.2008.26809 PMC2714374

[44] Razak F, Anand SS, Shannon H, Vuksan V, Davis B, et al (2007) Defining obesity cut points in a multiethnic population. Circulation 115: 2111–2118. 17420343 10.1161/CIRCULATIONAHA.106.635011

[45] Snehalatha C, Viswanathan V, Ramachandran A (2003) Cutoff values for normal anthropometric variables in asian Indian adults. Diabetes Care 26: 1380–1384. 12716792 10.2337/diacare.26.5.1380

[46] Stevens J, Truesdale KP, Katz EG, Cai J (2008) Impact of body mass index on incident hypertension and diabetes in Chinese Asians, American Whites, and American Blacks: the People's Republic of China Study and the Atherosclerosis Risk in Communities Study. Am J Epidemiol 167: 1365–1374. 18375949 10.1093/aje/kwn060 PMC2792196

[47] Bainey KR, Jugdutt BI (2009) Increased burden of coronary artery disease in South-Asians living in North America. Need for an aggressive management algorithm. Atherosclerosis 204: 1–10. 18980768 10.1016/j.atherosclerosis.2008.09.023

[48] Charbonneau-Roberts G, Saudny-Unterberger H, Kuhnlein HV, Egeland GM (2005) Body mass index may overestimate the prevalence of overweight and obesity among the Inuit. Int J Circumpolar Health 64: 163–169. 15945286 10.3402/ijch.v64i2.17969

[49] Garn SM, Leonard WR, Hawthorne VM (1986) Three limitations of the body mass index. Am J Clin Nutr 44: 996–997. 3788846 10.1093/ajcn/44.6.996

[50] Deurenberg P, Deurenberg Yap M, Wang J, Lin FP, Schmidt G (1999) The impact of body build on the relationship between body mass index and percent body fat. Int J Obes Relat Metab Disord 23: 537–542. 10375058 10.1038/sj.ijo.0800868

[51] Dulloo AG, Jacquet J, Solinas G, Montani JP, Schutz Y (2010) Body composition phenotypes in pathways to obesity and the metabolic syndrome. Int J Obes (Lond) 34 Suppl 2 S4–17. 21151146 10.1038/ijo.2010.234

[52] Gurrici S, Hartriyanti Y, Hautvast JG, Deurenberg P (1998) Relationship between body fat and body mass index: differences between Indonesians and Dutch Caucasians. Eur J Clin Nutr 52: 779–783. 9846588 10.1038/sj.ejcn.1600637

[53] Dulloo AG, Montani JP (2012) Body composition, inflammation and thermogenesis in pathways to obesity and the metabolic syndrome: an overview. Obes Rev 13 Suppl 2 1–5. 10.1111/j.1467-789X.2012.01032.x 23107254

[54] Gordon-Larsen P, Adair LS, Meigs JB, Mayer-Davis E, Herring A, et al (2013) Discordant risk: overweight and cardiometabolic risk in Chinese adults. Obesity (Silver Spring) 21: E166–174. 23505200 10.1038/oby.2012.152 PMC3486953

[55] Kadowaki T, Sekikawa A, Murata K, Maegawa H, Takamiya T, et al (2006) Japanese men have larger areas of visceral adipose tissue than Caucasian men in the same levels of waist circumference in a population-based study. Int J Obes (Lond) 30: 1163–1165. 16446744 10.1038/sj.ijo.0803248

[56] Misra A, Ganda OP (2007) Migration and its impact on adiposity and type 2 diabetes. Nutrition 23: 696–708. 17679049 10.1016/j.nut.2007.06.008

[57] Niu J, Seo DC (2014) Central obesity and hypertension in Chinese adults: A 12-year longitudinal examination. Prev Med 62c: 113–118. 10.1016/j.ypmed.2014.02.012 24552844

[58] Wang D, Li Y, Lee SG, Wang L, Fan J, et al (2011) Ethnic differences in body composition and obesity related risk factors: study in Chinese and white males living in China. PLoS One 6: e19835. 21625549 10.1371/journal.pone.0019835 PMC3098253

[59] Wang J, Thornton JC, Russell M, Burastero S, Heymsfield S, et al (1994) Asians have lower body mass index (BMI) but higher percent body fat than do whites: comparisons of anthropometric measurements. Am J Clin Nutr 60: 23–28. 8017333 10.1093/ajcn/60.1.23

[60] Anand SS, Tarnopolsky MA, Rashid S, Schulze KM, Desai D, et al (2011) Adipocyte hypertrophy, fatty liver and metabolic risk factors in South Asians: the Molecular Study of Health and Risk in Ethnic Groups (mol-SHARE). PLoS One 6: e22112. 21829446 10.1371/journal.pone.0022112 PMC3145635

[61] McKeigue PM, Shah B, Marmot MG (1991) Relation of central obesity and insulin resistance with high diabetes prevalence and cardiovascular risk in South Asians. Lancet 337: 382–386. 1671422 10.1016/0140-6736(91)91164-p

[62] Boyko EJ, Fujimoto WY, Leonetti DL, Newell-Morris L (2000) Visceral adiposity and risk of type 2 diabetes: a prospective study among Japanese Americans. Diabetes Care 23: 465–471. 10857936 10.2337/diacare.23.4.465

[63] Hayashi T, Boyko EJ, Leonetti DL, McNeely MJ, Newell-Morris L, et al (2004) Visceral adiposity is an independent predictor of incident hypertension in Japanese Americans. Ann Intern Med 140: 992–1000. 15197016 10.7326/0003-4819-140-12-200406150-00008

[64] Chandie Shaw PK, Berger SP, Mallat M, Frolich M, Dekker FW, et al (2007) Central obesity is an independent risk factor for albuminuria in nondiabetic South Asian subjects. Diabetes Care 30: 1840–1844. 17456841 10.2337/dc07-0028

[65] Lear SA, Humphries KH, Kohli S, Chockalingam A, Frohlich JJ, et al (2007) Visceral adipose tissue accumulation differs according to ethnic background: results of the Multicultural Community Health Assessment Trial (M-CHAT). Am J Clin Nutr 86: 353–359. 17684205 10.1093/ajcn/86.2.353

[66] O'Loughlin J, Maximova K, Tan Y, Gray-Donald K (2007) Lifestyle risk factors for chronic disease across family origin among adults in multiethnic, low-income, urban neighborhoods. Ethn Dis 17: 657–663. 18072375

[67] Rothman KJ (2008) BMI-related errors in the measurement of obesity. Int J Obes (Lond) 32 Suppl 3 S56–59. 18695655 10.1038/ijo.2008.87

[68] Stevens J, Juhaeri, Cai J, Jones DW (2002) The effect of decision rules on the choice of a body mass index cutoff for obesity: examples from African American and white women. Am J Clin Nutr 75: 986–992. 12036803 10.1093/ajcn/75.6.986

[69] Stevens J (2003) Ethnic-specific revisions of body mass index cutoffs to define overweight and obesity in Asians are not warranted. Int J Obes Relat Metab Disord 27: 1297–1299. 14574338 10.1038/sj.ijo.0802417

[70] Sebo P, Beer-Borst S, Haller DM, Bovier PA (2008) Reliability of doctors' anthropometric measurements to detect obesity. Prev Med 47: 389–393. 18619998 10.1016/j.ypmed.2008.06.012

[71] Stunkard AJ, Albaum JM (1981) The accuracy of self-reported weights. Am J Clin Nutr 34: 1593–1599. 7270483 10.1093/ajcn/34.8.1593

[72] Villanueva EV (2001) The validity of self-reported weight in US adults: a population based cross-sectional study. BMC Public Health 1: 11. 11716792 10.1186/1471-2458-1-11 PMC59896

[73] Health Canada.Canadian Guidelines for Body Weight Classification in Adults - Quick Reference Tool for Professionals. Available: http://www.hc-sc.gc.ca/fn-an/alt_formats/hpfb-dgpsa/pdf/nutrition/cg_quick_ref-ldc_rapide_ref-eng.pdf. Accessed 2013 April 25.

[74] Bes-Rastrollo M, Sabate J, Jaceldo-Siegl K, Fraser GE (2011) Validation of self-reported anthropometrics in the Adventist Health Study 2. BMC Public Health 11: 213. 21466678 10.1186/1471-2458-11-213 PMC3079646

